# Dynamic causal modelling of fluctuating connectivity in resting-state EEG

**DOI:** 10.1016/j.neuroimage.2019.01.055

**Published:** 2019-04-01

**Authors:** Frederik Van de Steen, Hannes Almgren, Adeel Razi, Karl Friston, Daniele Marinazzo

**Affiliations:** a Ghent University, Ghent, Belgium; b Monash Institute of Cognitive and Clinical Neurosciences and Monash Biomedical Imaging, Monash University, Clayton, Australia; c The Wellcome Centre for Human Neuroimaging, University College London, London, United Kingdom; d Department of Electronic Engineering, NED University of Engineering and Technology, Karachi, Pakistan

**Keywords:** Dynamic causal modelling, Resting state, EEG, Fluctuating connectivity

## Abstract

Functional and effective connectivity are known to change systematically over time. These changes might be explained by several factors, including intrinsic fluctuations in activity-dependent neuronal coupling and contextual factors, like experimental condition and time. Furthermore, contextual effects may be subject-specific or conserved over subjects. To characterize fluctuations in effective connectivity, we used dynamic causal modelling (DCM) of cross spectral responses over 1- min of electroencephalogram (EEG) recordings during rest, divided into 1-sec windows. We focused on two intrinsic networks: the default mode and the saliency network. DCM was applied to estimate connectivity in each time-window for both networks. Fluctuations in DCM connectivity parameters were assessed using hierarchical parametric empirical Bayes (PEB). Within-subject, between-window effects were modelled with a second-level linear model with temporal basis functions as regressors. This procedure was conducted for every subject separately. Bayesian model reduction was then used to assess which (combination of) temporal basis functions best explain dynamic connectivity over windows. A third (between-subject) level model was used to infer which dynamic connectivity parameters are conserved over subjects. Our results indicate that connectivity fluctuations in the default mode network and to a lesser extent the saliency network comprised both subject-specific components and a common component. For both networks, connections to higher order regions appear to monotonically increase during the 1- min period. These results not only establish the predictive validity of dynamic connectivity estimates – in virtue of detecting systematic changes over subjects – they also suggest a network-specific dissociation in the relative contribution of fluctuations in connectivity that depend upon experimental context. We envisage these procedures could be useful for characterizing brain state transitions that may be explained by their cognitive or neuropathological underpinnings.

## Highlights

• We describe fluctuating connectivity of rest-EEG in default and saliency network.
• Cross-spectral DCM was used to model effective connectivity.
• Parametric empirical Bayes was used to model connectivity fluctuations.
• Connectivity fluctuations show subject specific and common components.

## 1 Introduction

Evolution over time of segregated and integrated brain activity is somehow intrinsic to its definition in terms of distributed neuronal dynamics. On the other hand the genesis, nature, and the time scale of these changes are diverse, and represent an increasing focus of research. Structurally, the rewiring of white matter in humans – through axonal growth or pruning – is unlikely to occur during late development (i.e. between age 2 and 18); however, changes in white matter tracts, such as increased (possibly activity dependent) myelination and axonal diameter have been shown by several studies (e.g. Giedd et al., 1999; Paus, 2010; Rademacher et al., 1999). Together with these structural changes, long and short range functional connectivity (FC) also changes during development (Hagmann et al., 2010). Here, FC is defined as the statistical dependencies among observed neurophysiological measures (e.g. correlation between blood oxygen level dependent (BOLD) signals; Friston, 2011). More recently, changes in FC at a shorter time-scale have been investigated (e.g., Allen et al., 2014; Chang and Glover, 2010; Vidaurre et al., 2017; Wang et al., 2016). The time scale of these studies is on the order of minutes (i.e. during a typical resting-state functional magnetic resonance imaging (rs-fMRI) protocol) – as opposed to the developmental studies which are in the order of years. Typically, the dynamic FC is quantified using a (sliding) window approach: the resting state time-series are segmented into (partially overlapping) windows and FC is calculated for each window. This approach allows researchers to assess the trajectory of FC over time for different networks/states. Alternatively to sliding windows, Vidaurre et al. (2017) showed, using a hidden Markov model, that the transitions between networks (states) are non-random. Also, time-varying connectivity has been studied using adaptive multivariate autoregressive models - by means of e.g. Kalman filtering approach, generalized recursive least square algorithm or sliding-windows - which quantifies fluctuations in directed functional connectivity (Arnold et al., 1998; Astolfi et al., 2006; Ding et al., 2000; Milde et al., 2010). In addition, an approach using a Bayesian framework to extract, from the data, the parameters accounting for fluctuations in the joint dynamics of coupled oscillatory systems has been developed in (Stankovski et al., 2012), and recently applied to investigate time-varying phase coupling in EEG (Stankovski et al., 2017). Note that two recent studies (Heitmann and Breakspear, 2017; Liégeois et al., 2017) also made the important point of distinguishing the meaning of “dynamic” from the statistical point of view (i.e., non-uniformity in time) from the link of these fluctuations to the intrinsic dynamics of neural populations that generate the recordings under examination.

However, studies dealing with fluctuations in (directed) functional connectivity cannot directly provide evidence of fluctuations in the underlying effective connectivity – which is defined as the directed (causal) influence one neuronal population exerts on the other. Effective connectivity can only be inferred using a forward or *generative model* of how activity in one brain region, or external stimulus, affects activity (i.e. causes a change in activity) in another brain region. Since we usually do not measure neural activity directly, the generative model usually includes a forward model of how (hidden) neural activity is mapped to the (observed) measurement (Friston, 2011). Inferring effective connectivity therefore requires the inversion of this generative model. This can be done with standard (variational) Bayesian model inversion, as in dynamic causal modelling (DCM; David et al., 2006; Friston et al., 2012, 2003).

Several studies have investigated fluctuations of effective connectivity obtained from electrophysiological recordings (Cooray et al., 2015; Papadopoulou et al., 2015, 2017) and fMRI (Park et al., 2017) using DCM. These electrophysiological studies were conducted in the context of tracking connectivity changes around periods of epileptic events, while the fMRI study investigated healthy subjects during rest. Cooray et al. (2015) used Bayesian belief updating, in which between window differences were modelled as a random walk. This contrasts with the approach in Papadopoulou et al. (2015), where the authors used a set of temporal basis functions to model connectivity fluctuations. In this approach, all windows were inverted simultaneously, making it computationally expensive. In the current work, a hierarchical model is employed: each window is inverted independently and subsequently, fluctuations are modelled over windows; i.e., at a between-window (within-subject) level. More specifically, a Bayesian linear model with temporal basis functions is used to model between window-differences (Papadopoulou et al., 2017; Park et al., 2017). Parametric empirical Bayes (PEB, Friston et al., 2016) is the procedure used to estimate this second level model. The main difference between Papadopoulou et al. (2017) and Park et al. (2017) is that the latter used multilevel PEB to make inferences at the group level, while the former concatenated the data from different subjects at the group level. In this paper, we use the same technology as described in Park et al. (2017) to assess fluctuations in effective connectivity at the within and between-subject level, with a special focus on systematic, time sensitive fluctuations that may be conserved over subjects.

In this work, we used eyes-open resting state electroencephalographic data (EEG) time series from healthy subjects. Our goal was to quantify how effective connectivity fluctuates over time using hierarchical Bayesian modelling. More specifically, we investigate whether connectivity fluctuates in a subject-specific manner and/or whether there are systematic components embedded within the connectivity trajectories that are conserved over subjects. We addressed this by using DCM for cross spectral density data features (CSD) combined with (multilevel) PEB (Friston et al., 2012, 2016). DCM was used to infer effective connectivity from windowed resting-state EEG time-series. Then, PEB was employed to characterize the trajectory of DCM parameters during the 1 min recording session. Applying the approach described in Park et al. (2017) to EEG allowed us to track fluctuations in DCM connectivity parameters on the time-scale of seconds and minutes. It is important to note that the generative model (i.e., DCM) for electromagnetic time series is biophysically more detailed compared to DCM for rest fMRI: the hidden states in DCM for CSD applied to EEG consist of voltages and firing rates of specific neuronal cell populations, while in fMRI the hidden states are an abstraction of neural activity (Friston et al., 2014; but also see recent developments Friston et al., 2017).

Our hypothesis was that we would be able to detect within subject (between window) fluctuations – in all subjects – in key (intrinsic) brain networks and, crucially, some of these fluctuations would have predictive validity in the sense that they would show systematic time effects in relation to the onset of the recording session. To address this hypothesis, we therefore analysed a large (publicly available) cohort of data, paying special attention to the hierarchical (PEB) modelling of random effects at the within-window, within-subject and within-group levels respectively. This analysis is offered as a proof of principle that systematic aspects of dynamic effective connectivity can be recovered from EEG data. We anticipate that the methods described below may be usefully applied to test for experimental effects on dynamic connectivity and implicit short-term synaptic plasticity.

## 2 Methods

### 2.1 Data and pre-processing

In this study, we used 1 min eyes open EEG recordings from the EEG Motor Movement/Imagery PhysioNet dataset (Goldberger et al., 2000; Schalk et al., 2004). The data was acquired using the BCI2000 system (www.bci2000.org). The EEG channels were placed on the scalp according to the international 10-10 system (Chatrian et al., 1985). The data was provided in EDF+ format, containing 64 EEG channels, each sampled at 160 Hz. In total, 109 subjects participated in the experiment.

The data were pre-processed using EEGLAB running on MATLAB (Delorme and Makeig, 2004). The 60Hz power line noise was first removed using the Cleanline EEGLAB plugin. Afterwards, the data was high-pass filtered using default settings, with a lower-cutoff of 1Hz. Then, a low-pass filter with high-cutoff of 45 Hz and default settings was applied. Periods of data contaminated with blink artefacts were repaired using independent component analysis. Bad channels were removed based on visual inspection. Finally, the data was average-referenced.

### 2.2 Dynamic causal modelling

The pre-processed data was imported in SPM12 (Wellcome Trust Centre for Human Neuroimaging; www.fil.ion.ucl.ac.uk/spm/software/spm12). DCM for CSD was used for further analyses (DCM12, Friston et al., 2012). In brief, DCM explains the observed CSD by combining a generative (biophysically plausible) neural mass model and an observation model that maps neuronal states to the observed data. Each electromagnetic source or node is equipped with three neuronal subpopulations: pyramidal cells, inhibitory interneurons and spiny stellate cells (the ‘ERP’ model; Moran et al., 2013). The connections between subpopulations within a node are termed intrinsic connections, while connections between nodes are termed extrinsic connections. There are three types of extrinsic connections: forward, backward and lateral connections, which differ in terms of their origin and target subpopulation. The type of connection can be determined based on the hierarchical organisation of the cortex (Felleman and Van Essen, 1991). The generative model thus describes the dynamics of the (hidden) neural states. Using variational Bayesian methods, we can invert the generative model in order to obtain posterior density estimates of the parameters. These estimates are of interest and used for further inferences. In short, the difference between DCM and the classical approach of estimating cross spectral densities at the EEG electrodes is that we are trying to find neuronal parameters that produce the (hidden) neural dynamics, which in turn give rise to observed (complex) cross spectral densities. Note that the use of complex cross spectra retains all the information about correlations over different time lags – in the time domain (unlike simple analyses of functional connectivity at zero lag). The default forward model provided by SPM12 was used for the observation equation (which is in essence a linear mapping from the hidden neural states to the EEG sensor spectral densities; i.e., a conventional lead field or gain matrix) that embodies volume conduction.

**Fig. 1:**
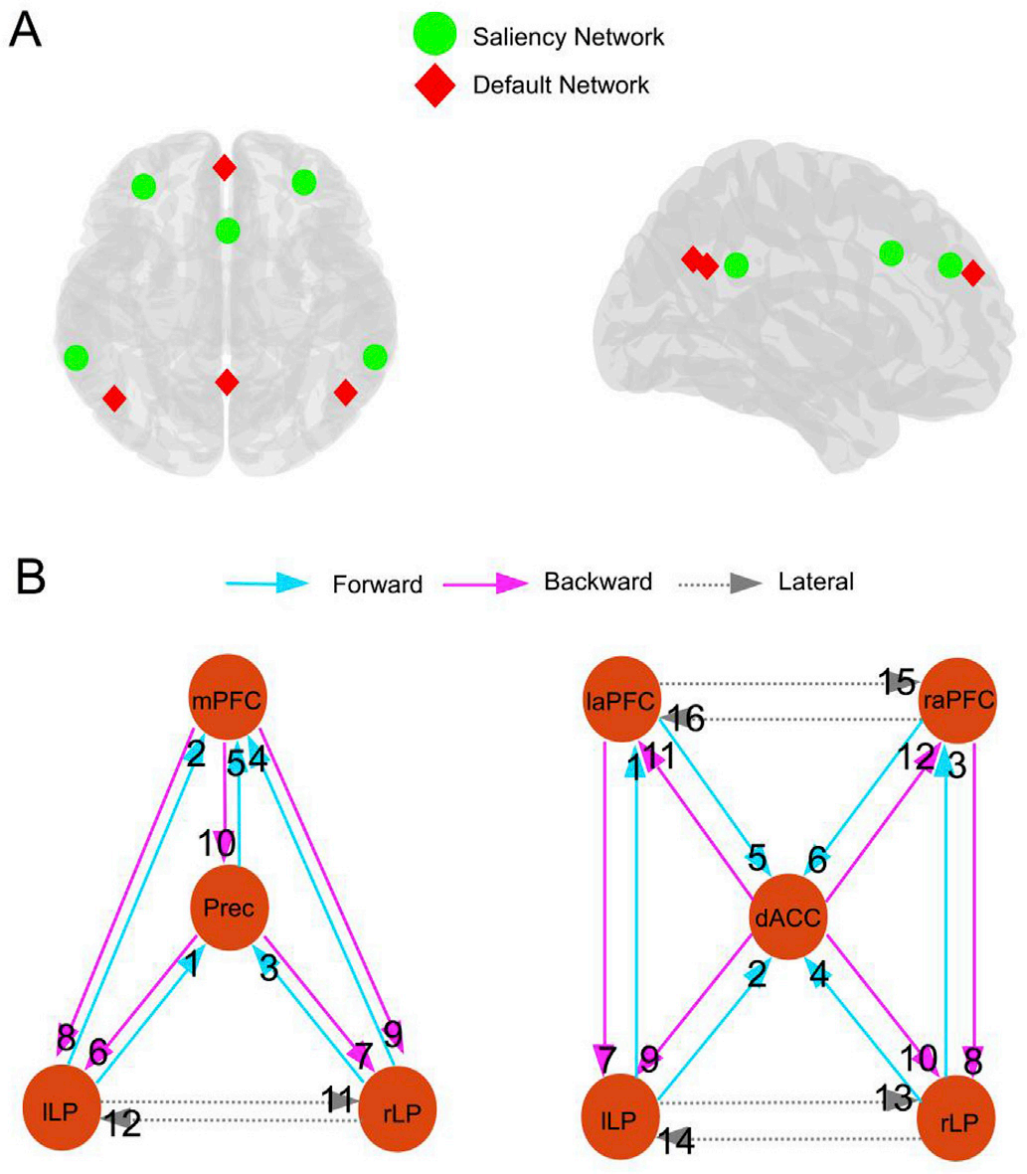
The default and saliency network studied in this paper are shown. Panel A shows the locations of sources included in both network. The default mode network included bilateral parietal areas (l/rLP), medial prefrontal cortex (mPFC) and the precuneus (Prec). The saliency network included bilateral parietal areas (l/rLP), dorsal anterior cingulate cortex (dACC) and bilateral anterior prefrontal cortex (aPFC). Panel B shows a schematic representation of the fully connected model in both networks. Light blue arrows depict forward connections, black arrows depict backward connections, and grey arrows depict lateral connections. The left panel corresponds to the default network while the right panel represents the saliency network. The numbers are connectivity codes used in subsequent plots.

In DCM for EEG, the anatomical locations of the nodes need to be specified *a priori*. We inverted, two (fully) connected models (hierarchical inversion, see below), one for the default mode network and one for the saliency network. For the default network, the following four nodes were chosen: left and right lateral parietal area (l/rLP; MNI coordinates: −46 −66 30; 49 −63 33, respectively), posterior cingulate/Precuneus (Prec; MNI coordinates: 0 −58 0) and medial prefrontal cortex (mPFC; MNI coordinates: −1 54 27, see Fig. 1A). For the saliency network, left and right lateral parietal (l/rLP; MNI coordinates: ±62 -45 30) area, left and right anterior prefrontal cortex (l/raPFC; MNI coordinates: −35 45 30; 32 45 30) and dorsal anterior cingulate cortex (dACC; MNI coordinates: 0 21 36 see Fig. 1A) were specified. These nodes were chosen based on the sources used in (Razi et al., 2017). In both networks, the nodes were connected with forward, backward and lateral connection as described by (David et al., 2006; David et al., 2005; Felleman and Van Essen, 1991). See Fig. 1B for a schematic presentation of the presumed coupling among the sources. Each node was treated as a patch on the cortical surface (‘IMG’ option in SPM12). As a diagnostic check, we calculated the explained variance of each DCM. In the supplementary materials, the mean (across subjects) explained variance as a function of window is shown, which shows that the DCM are able to fit the observed CSD's well.

### 2.3 Time-varying DCM using parametric empirical Bayes

To characterize dynamic effective connectivity, the resting state EEG time-series (1- min duration) were divided into 1- sec consecutive epochs (Papadopoulou et al., 2015, 2017). For each time-window, two DCM's (one for each network) were fitted to the data as described above. Then, the between-window differences within a network were modelled using a second level (general) linear model with a set of temporal basis functions (i.e., a discrete cosine set and a mono-exponential decay function) as regressors. More specifically, a hierarchical generative model was employed in which first level (i.e. window-level) DCM parameters are treated as a linear mapping from a second level model (i.e. subjects-level):

(1) $$\theta =\left(X⊗I_{B}\right)\beta +\varepsilon$$

(2) β=η+ξ

Here, *θ* are the vectorised (multivariate) DCM parameters. More specifically, the first Z = 1,...,B elements of *θ* are the DCM parameters of the first window. The vectorised DCM parameters are thus stacked window-wise with B equals the number of DCM parameters and I_B_ is the identity matrix of size B. W equals the number of windows so that ${\theta =\left[\theta_{11},...,\theta_{1B},...,\theta_{W1},...,\theta_{WB,}\right]}^{T}$. ***X*** refers to the second level design matrix and is of size W x P. The first column is a constant term while the other columns are temporal basis functions.

**Fig. 2:**
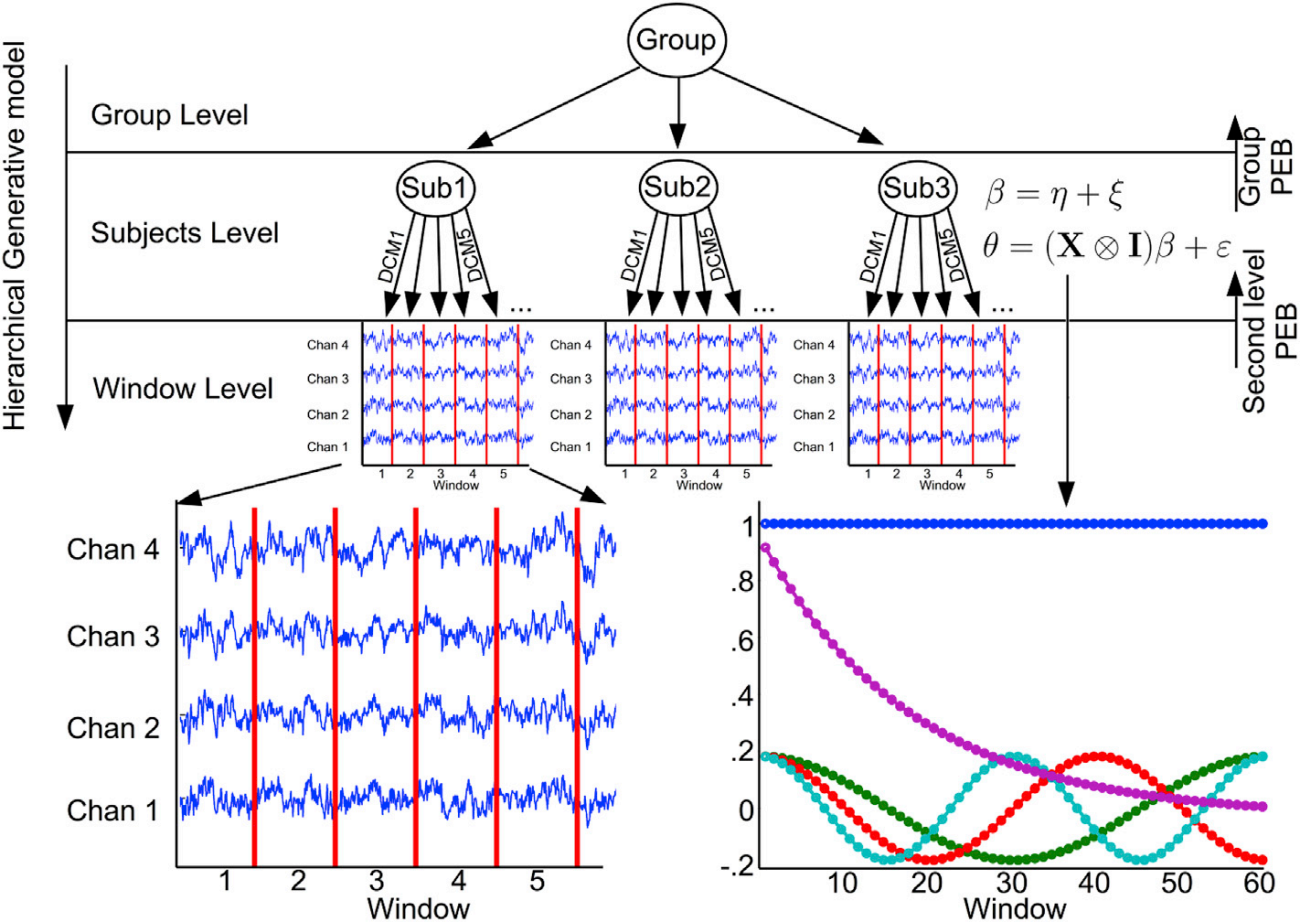
The hierarchical generative model used in this work. The lowest level depicts the window-level. At this level, two DCMs – one for the default mode network and one for the saliency network – were inverted for each window. Using parametric empirical Bayes (PEB), time dependent differences between windows are modelled at the within-subject level. The temporal basis functions used to model these differences are shown in the lower right panel. At the group level, the mean of time-dependent changes in effective connectivity was assessed, using PEB of between-subject effects (group-PEB).

In this work we specified four temporal basis functions. These include a discrete cosine transformation (DCT) basis set, and one mono-exponential decay basis function. The DCT functions were defined as $\sqrt{\frac{2}{W}}cos\left(\frac{\pi }{W}\left(n+\frac{1}{2}\right)k\right)$, n = 0,…,W-1 and k = 2,..,4. The exponential decay function was defined as $e^{-\frac{n}{16}}$ (see Fig. 2). This second level design matrix describes how the DCM parameters change over time and thus contains 5 columns (P = 5). The Kronecker tensor product (⊗) of ***X*** and ***I***_*B*_ (the identity matrix of size B) means that each DCM parameter can show one or more second level effects. Furthermore, β are the second level parameters represented as a column vector of length B x P. The last term in (1), ε, is the inter-window variability (i.e., random effects) and ξ corresponds to the random fluctuations in the second level parameters. Using PEB, the parameters in (1) and (2) can be estimated for each subject (i.e., subject-specific PEB) from the prior and posterior means and covariances from each window as described in (Friston et al., 2016). The full hierarchical model is thus defined as

$$\ln\;p\left(y,\theta ,\beta \right)=\overset{i=1:W}{\underset{i}{\sum }}\ln\;p\left(y_{i}\text{|}\theta \right)+\ln\;p\left(\theta \text{|}\beta \right)+\text{ln}p\left(\beta \right)$$

$$p\left(y_{i}\text{|}\theta \right)∼\text{Ν}(\text{Γ}\left(\theta \right),\Sigma_{i})$$

$$p\left(\theta \text{|}\beta \right)∼\text{Ν}\left(\left(X⊗I_{B}\right)\beta ,\Sigma \right)$$

(3) p(β)∼Ν(η,Ξ)

This model may look complicated; however, it provides a relatively straightforward and generic model for any hierarchical inference with Gaussian (i.e., parametric) random effects. The second level of this model is effectively a general linear model, while the first level can be any nonlinear (and dynamical) model.

The first line in (3) describes the full joint probability density of the data (within-window), first and second level parameters. The second line describes the likelihood of the data where Γ(θ) is a (non-linear) mapping (i.e. DCM) from first level parameters to observed responses in the ith window and $\Sigma_{i}$ is the covariance of the observation noise (assumed to be zero mean). The third line specifies the conditional probability of the first level parameter given the second level parameters. Due to the dependency upon second level parameters, this is also called an empirical prior. The covariance matrix is parametrized using a single precision parameter γ: $\Sigma^{-1}=I_{w}⊗\left(Q_{0}+e^{-\gamma }Q_{1}\right)$ with $Q_{1}=16^{-1}\mathrm{pC}$ and pC is the prior covariance of the DCM parameters when estimated using non-empirical priors (i.e. during conventional estimation of the DCM here $\mathrm{pC}=16^{-1}I_{B}$). The last line in (3) specifies the distribution of the second level parameters with mean η and covariance Ξ. Zero mean and $\frac{I_{P}}{\left\|X\right\|}⊗pC$ priors where used for η and Ξ In this work, the second level models were estimated for the forward, backward and lateral connections separately – to avoid over-parametrization of the model. The hierarchical model above can be inverted using standard variational Bayes (e.g., Variational Laplace) to provide posterior densities over the model parameters at all levels.

Once the second level parameters are estimated, Bayesian model reduction can be used to efficiently estimate reduced second level models, from the full second level model (see Friston and Penny, 2011; Friston et al., 2016, for more details). Here, we derived all combinations of the four temporal basis functions – so that for each subject, 16 reduced models were estimated from the full second level model (using spm_dcm_bmc_peb.m). Bayesian model comparison of those second level models was conducted by summing the log evidences for each second level model over subjects.

Finally, the second level parameter estimates (i.e., posterior mean and covariance), from the second level model were entered into a third level (PEB) model for group level inference. Specifically, subject-specific parameters of between-window effects for each DCM parameter were modelled with a second level model but now, the design matrix contained only a constant term. In other words, the average of – or conserved – between-window effects across subjects were modelled (we refer to this as the group-PEB). This enabled us to ask which between-window effects of specific connections are conserved over subjects. Finally, any parameters of the group-PEB that do not contribute to the log evidence were pruned away, using Bayesian model reduction and a greedy search (implemented in spm_dcm_peb_bmc.m, see Friston and Penny, 2011 for more information about the greedy search algorithm).

To preclude local minima at the first (within-window) level, we used PEB scheme for estimating the within-window DCM parameters. In this application of PEB, the first level parameters are iteratively re-estimated under the assumption they are generated from a between-window mean (using spm_dcm_peb_fit.m). This uses empirical shrinkage priors to pull solutions away from local minima (Friston et al., 2015). Since our study involved inverting a large number of DCMs, the spm_dcm_peb_fit.m code was compiled as a stand-alone executable (using the MATLAB mcc command). This enabled the initial estimation to be conducted in parallel on the Ghent University High Performance Computing infrastructure. In order to check the robustness of the results, we randomly split the group into 2 subgroups and performed the second level BMC and group-level PEB separately for these subgroups.

In summary, Bayesian model reduction (i.e., selection of reduced models) of second level (subject-specific) models was used to identify the temporal basis functions that best explained between-window differences. The group-PEB analysis was then performed to assess which time-dependent components are conserved over subjects.

## 3 Results

**Fig. 3:**
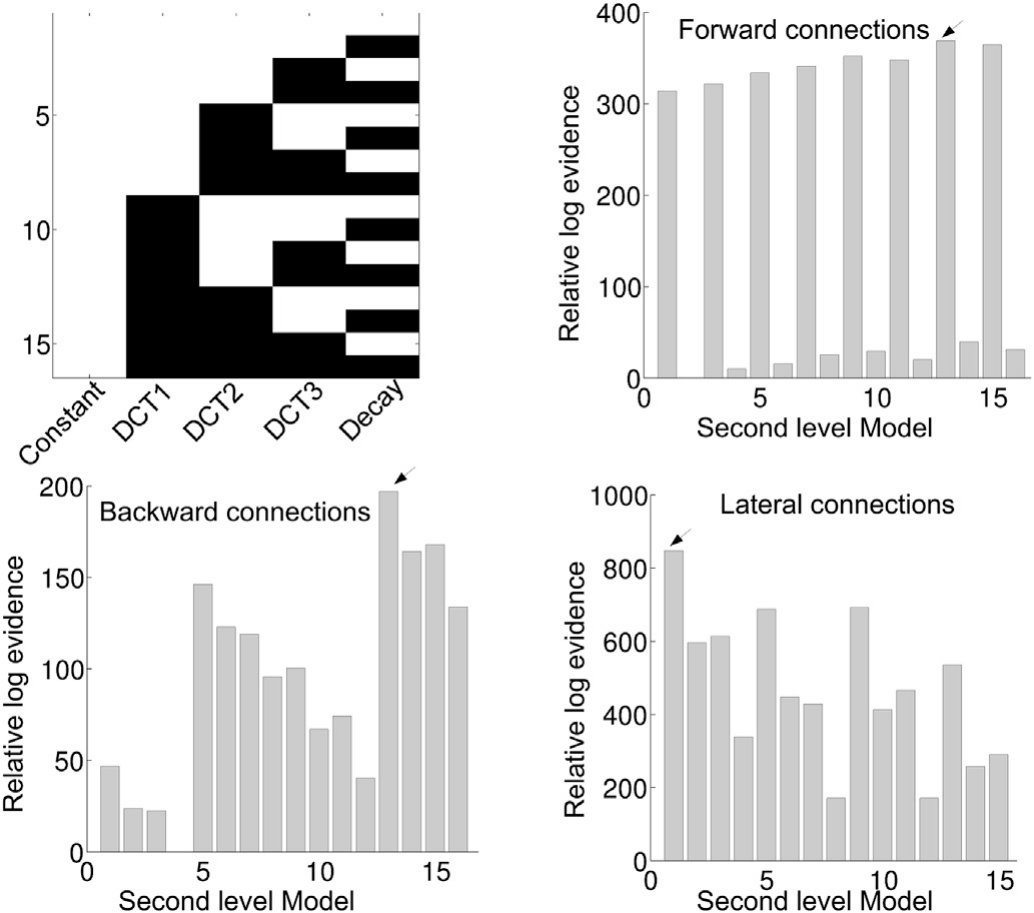
The results for the second level (between-window, within-subject) Bayesian model comparison for the default mode network are shown. The top left panel shows the second level model space. These included all combinations of second level basis functions. The constant term was included in all reduced models. The top right panel shows the relative log evidences of all second level models for the forward connections. The bottom left and bottom right panels show the relative log evidences of second level models for the backward and lateral connections respectively. The arrow indicates the model with the highest model evidence.

**Fig. 4:**
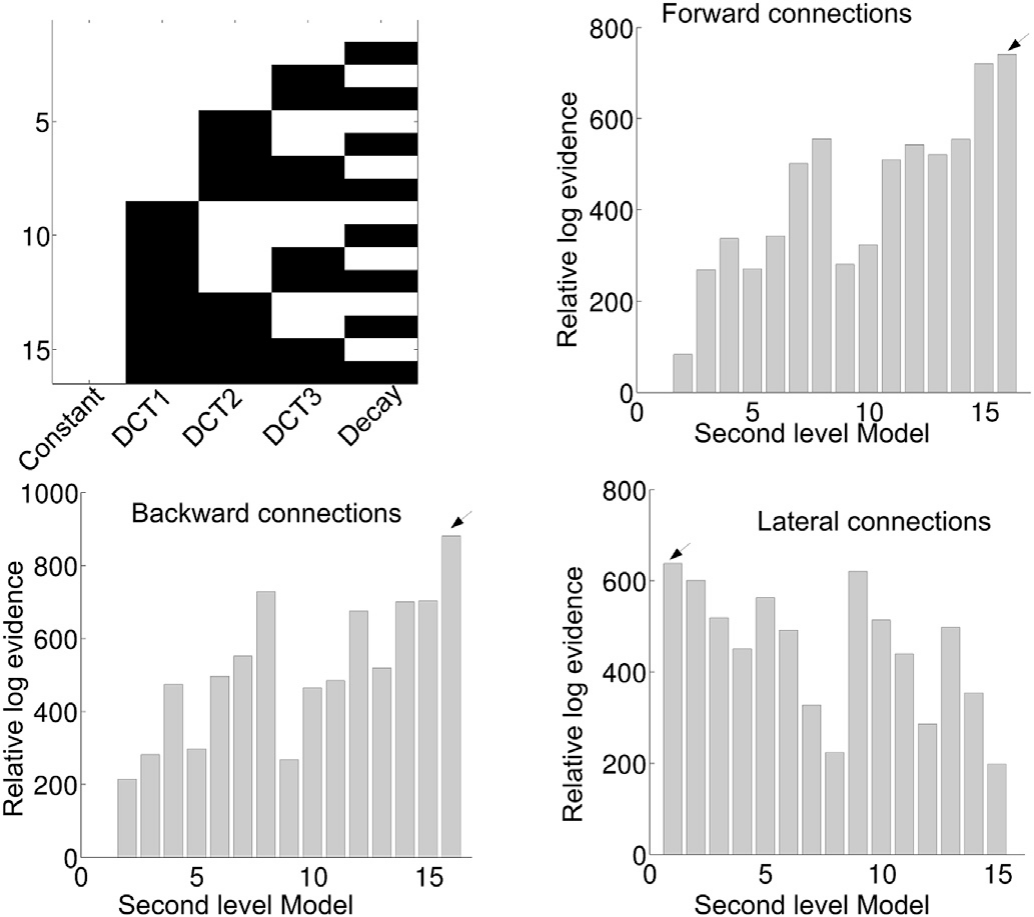
Same format as for Fig. 3. but now for the saliency network.

Our goal was to estimate time-dependent changes in effective connectivity using rest-EEG. More specifically, we wanted to characterize the connectivity trajectories that may or may not be conserved over subjects. Segmented data was analysed with DCM for CSD combined with (multilevel) PEB. We first estimated a full second level PEB model for each subject. Then using Bayesian model reduction, all combinations of second level regressors (except the constant, which was always included in the model) were derived from the full model (see Fig. 3 for the second level model space). The most likely combinations of regressors were detected by taking the sum of the free energies over subjects, for all combinations of second level basis functions. Model 16 is the model with only a constant term. This is thus the ‘null model’ which models random fluctuations around a constant. The results are shown in Fig. 3, Fig. 4 for the default mode and saliency network respectively. For the default mode network, we see that the evidence for the null models is much lower (i.e. the log-scale difference is larger than 3, which can be a considered as very strong evidence) compared to the winning model for each of the connection types. The winning model seems to be connection type specific. For the forward and backward connections the evidence points to model 13, which contains DCT3 and the decay basis functions. For the lateral connections, all basis functions (i.e. model 1) appear to be relevant for explaining between window differences. For the saliency network, we only found evidence against the null model for the lateral connections as can be seen in Fig. 4. Here, the evidence was in favour of model 1 (all basis functions).

Next, we performed a group-PEB of second level effects to investigate which DCM parameter(s) show one or more time dependent effects that are conserved across subjects. This means that for the group PEB, there are 5 (basis functions + constant) by 5 (forward connections), 5 (backward connections) or 2 (lateral connections) parameters for the default network and 5 by 6 (forward connections), 6 (backward connections) or 4 (lateral connections) parameters for the saliency network. Note that we only modelled extrinsic connections. These are parameterized in terms of log gains. This way, the connections are constrained to be excitatory (extrinsic or between source cortico-cortical connections in the brain are excitatory or glutamatergic). In the neural model, intrinsic connectivity is modelled with four between subpopulations couplings (for each source separately), where contextual changes in intrinsic coupling are modelled by a gain on the amplitude of the synaptic kernels. In effect, this parameterization controls the populations sensitivity to afferent inputs (Kiebel et al., 2007). For simplicity, we only considered fluctuations in extrinsic connectivity. The group-PEB was conducted for the forward, backward and lateral connections separately (as was done for the second level models).

**Fig. 5:**
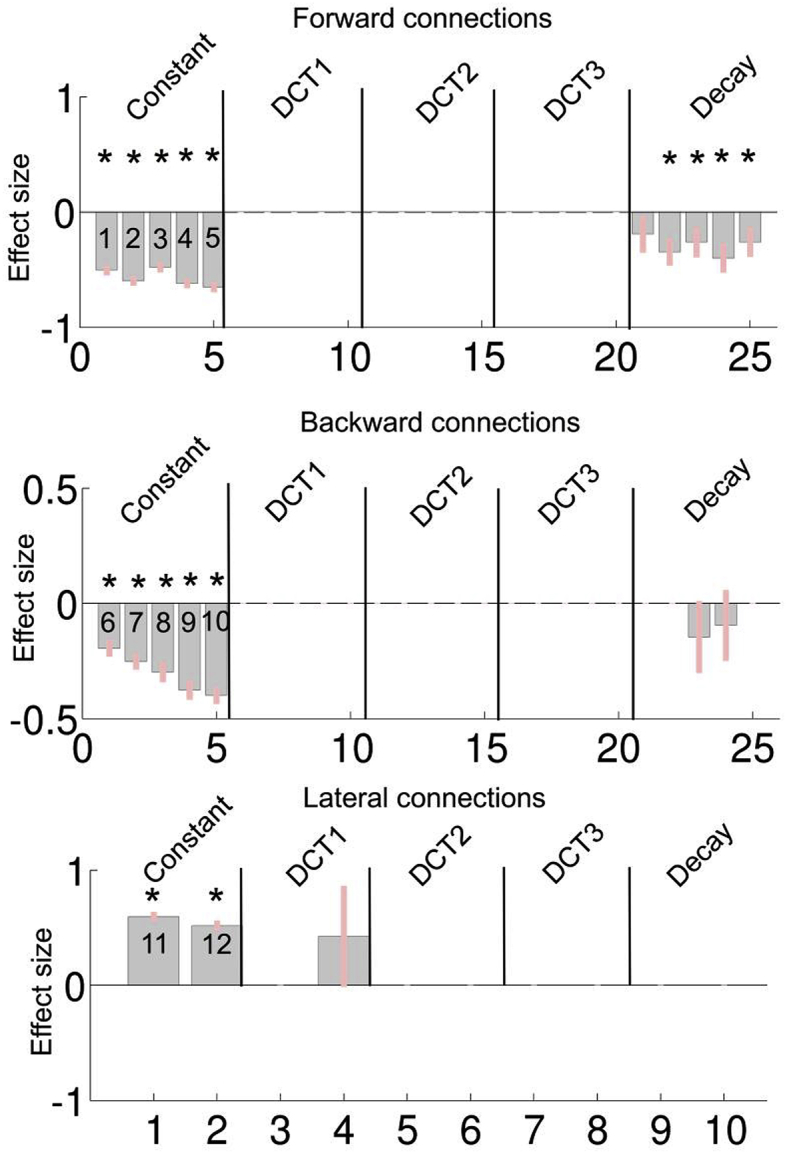
The results of the group PEB, following BMA for the default mode network. The numbers inside the bars are the connectivity codes corresponding to the numbers given in Fig. 1. The vertical lines separates the parameters (i.e. β in (1)) based on the second level basis functions, in which the ordering of the connections is the same as for the constant term. The top, middle and bottom panel shows the results for the forward backward and lateral connection respectively. Parameters with *, have a posterior probability (Pp) > .95.

**Fig. 6:**
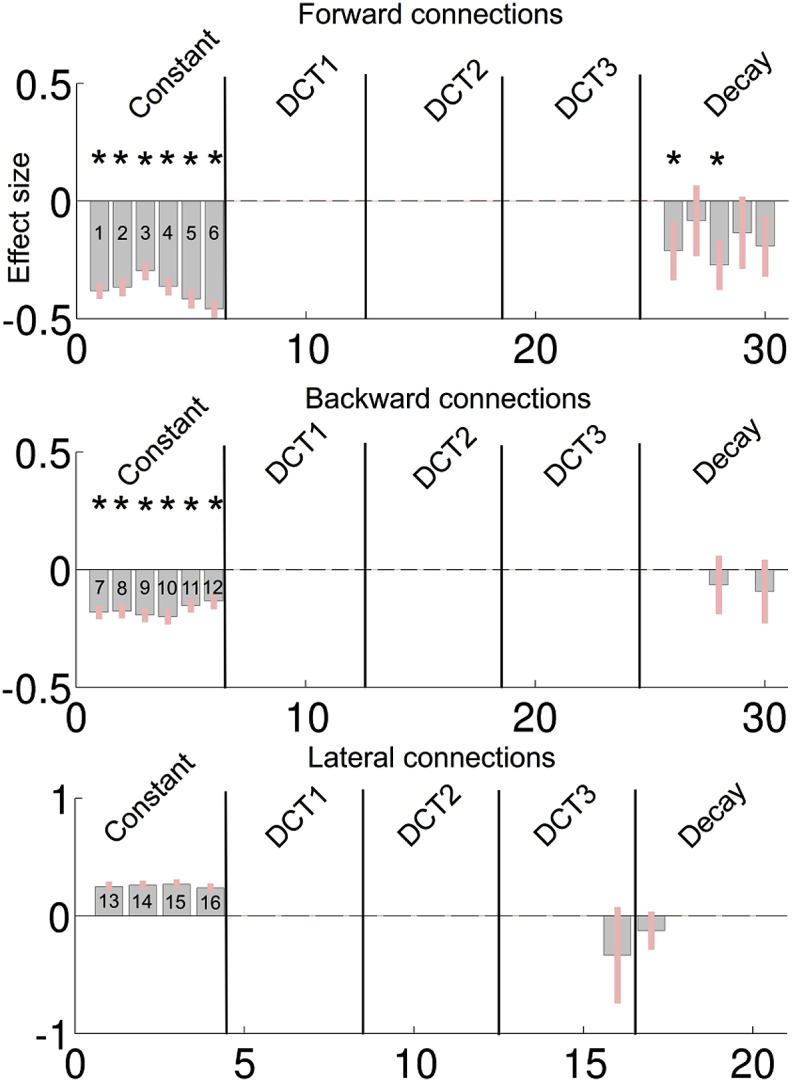
Same format as for Fig. 5 but now for the saliency network.

**Fig. 7:**
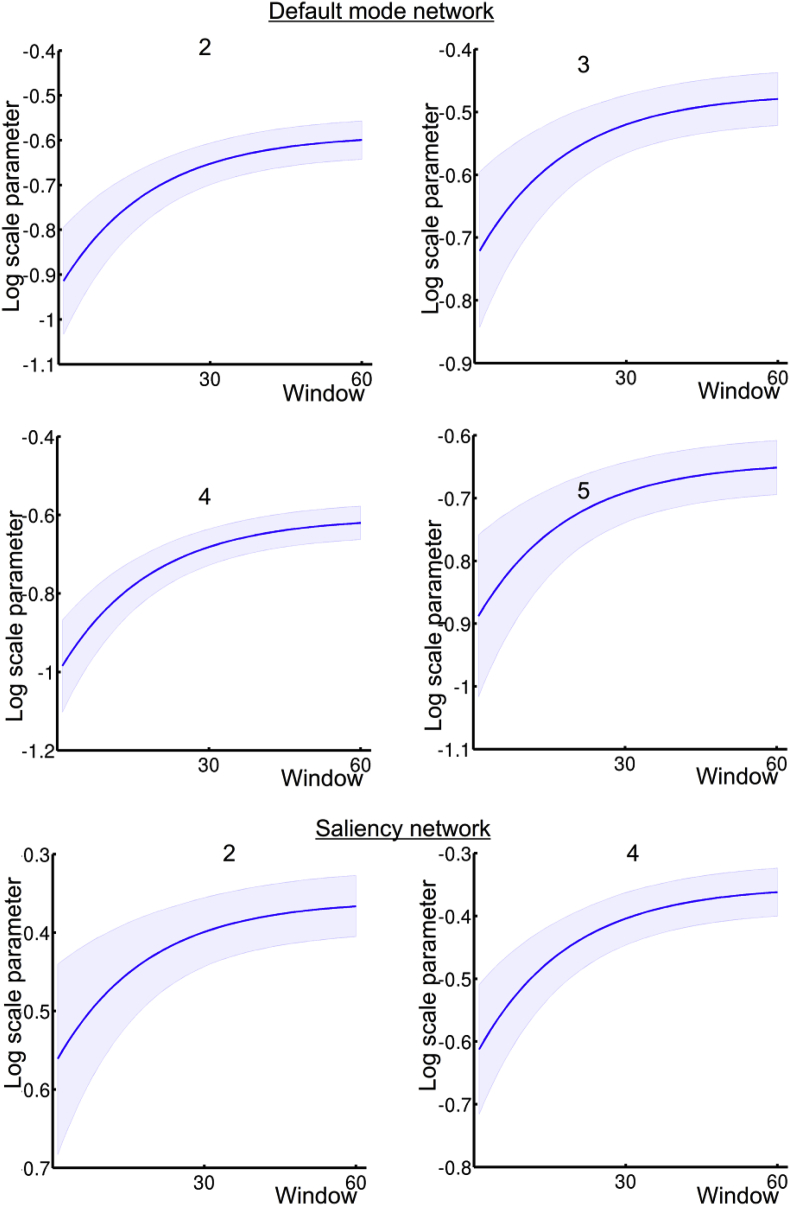
Predicted DCM (log scale) parameter trajectories for the default network are shown in the top two panels and for the saliency network in the bottom panels. The blue shaded areas around the lines indicate a 90% confidence interval (i.e., Bayesian credible interval) of the predicted trajectories for each connection at the group level. Connectivity codes are explained in the caption of Fig. 1.

Following the inversion of the group PEB, a greedy search was performed to prune any parameters that do not contribute to the log evidence (i.e., parameters that increase complexity without increasing accuracy). The search algorithm used Bayesian model reduction (BMR) to remove redundant connection parameters from the full model; until there was no further improvement in model-evidence (Friston and Penny, 2011). The posterior densities of the parameters of the best 256 models from this search procedure were then averaged, weighted by their model evidence (i.e. Bayesian Model Averaging, BMA). This procedure was performed for both networks. The results for the default mode network and the saliency network are shown in Fig. 5, Fig. 6 respectively. From Fig. 5, it is clear that the DCT components for the DMN were either pruned away during the greedy search or that the posterior probability (Pp) was below 0.95. More specifically, only for the first DCT component, one lateral connection survived pruning. Interestingly, only the constant term and the monotonic decay basis function showed a clear effect. For the monotonic decay, the forward connections and the lateral connections showed a negative effect (resulting in monotonic increasing trajectory). The monotonic increase was most pronounced for the forward connections (4 out of 5 connections showed a Pp > .95). In order to demonstrate the time dependency effect on effective connectivity parameters more clearly, the (posterior predictive expectations of the) group level trajectories for each of the DCM parameters are shown in Fig. 7. Only the trajectories of connectivity parameters with at least one basis function component with Pp > .95 are shown which are connections 2, 3, 4 and 5 (forward connections) for the default mode network.

In Fig. 6, the group PEB parameters after BMR and BMA are shown for the saliency network. For the decay component, a similar pattern was observed: forward connections showed a negative monotonic decay effect. With respect to the DCT components, only one survived Bayesian model reduction, however Pp < .95. In Fig. 6, the predicted trajectories of connection 2 and 4 are shown.

The results for the split-group analysis are reported in the supplementary materials. These results are largely consistent with the whole group results.

## 4 Discussion

In this work, we build upon recent characterizations of dynamic brain connectivity at rest (e.g. Allen et al., 2014; Chang and Glover, 2010; Vidaurre et al., 2017; Wang et al., 2016). Here, we used the approach proposed recently by Park et al. (2017). Parametric empirical Bayes (PEB) was employed to model fluctuations in effective connectivity; where effective connectivity was inferred using dynamic causal modelling (DCM). One minute rest-EEG time-series were segmented into 60 non-overlapping windows. Then DCM for cross spectral densities was (iteratively) fitted to the data for each window. Afterwards the differences in DCM parameters between the windows were modelled with a second level linear model. The design of this second level comprised of a number of temporal basis functions plus a constant term. This way time dependent fluctuations in effective connectivity were modelled. Here, we used 3 DCT components and one exponential decay component – whose linear combinations can cover a wide range of plausible trajectories. This hierarchical model was estimated with PEB. Note that, in this (EEG) study, changes in effective connectivity were investigated at much faster time scales compared to fMRI.

A major advantage of PEB is that deep hierarchical models can be inverted recursively bypassing posterior densities to subsequent levels (i.e., PEB of PEB). This allowed us to ask which components of the parameter trajectories are conserved over subjects. Another advantage of PEB is that reduced second (or third) level models can be derived efficiently from a full model, without having to re-estimate the reduced lower levels. Moreover, the estimation of first level models can be conducted in parallel – and requires less memory for processing. This offers a computational advantage over other approaches that only admit parallelization over subjects but not windows (Cooray et al., 2015; Papadopoulou et al., 2015). For comparison, we tried the approach described in Papadopoulou et al. (2015) to our data but were unable to estimate the models because of insufficient RAM (64 GB were available). DCM provides a posterior estimate of the noise precision matrix, which needs to be inverted by the variational Bayesian inversion scheme. In case of 60 windows and 64channels, this matrix is too big to be inverted using 64 GB of RAM. In the approach used here, the inversion of a single DCM took about 10–15min – so that in total about 100 days would be needed if estimated consecutively. Because we conducted the estimation in mass-parallel, the inversion of all DCM's took less than 2 days.

Bayesian model comparison of the second level (between-window, within-subject) models showed that for the default mode network, the evidence of the winning models for the forward, backward and lateral connections were higher than the ‘null’ model. For the saliency network, evidence for the ‘null’ model was highest for forward and backward connection but not for the lateral connections. Thus, we provide evidence that effective connectivity parameters change systematically over time, even over the course of 1 min using EEG. In other words, the observed differences between windows cannot be explained by non-systematic random fluctuations around a constant; when comparing the log evidence for second level models (which embodies a trade-off between model accuracy and model complexity).

The variance explained has an interesting interpretation in the context of DCM for cross spectral responses. The results in (supplemental) Fig. S5 suggest that differences in variance explained over time are small in relation to the differences between networks. Although quantitatively small, the decrease in variance explained over time is evident for both networks. The explanations for these systematic effects are subtle and may call for further Bayesian model comparison to fully characterise. This is because the variance explained in dynamic causal modelling of spectral data does not reflect the level of observation noise or random fluctuations. This follows because observation noise is modelled explicitly as a spectral (i.e., covariance) component during model inversion. This means that differences in accuracy reflect differences in the capacity of various spectral or covariance components to explain the observed spectra (as opposed to changes in signal-to-noise or artefacts). In turn, this suggests that it should be possible to use model comparison to identify the form and source of these systematic effects, using suitably augmented DCMs. More generally, much work has been done regarding the effects of artefacts on resting-state fMRI functional connectivity (Murphy et al., 2013). However, for EEG the situation is less clear. We have shown in a recent paper that DCM in general is relatively robust to preprocessing strategies such as global signal regression – GRS (Almgren et al., 2018). More specifically, the influence of GRS on DCM estimates, showed to have minimal effect on the connectivity parameters. Nevertheless, future work should investigate the effects of artefacts and confounders on both effective and functional connectivity estimates in EEG.

Comparing the results for the default mode network with the results from Park et al. (2017) that used resting state fMRI, we see that in their analysis, all DCT components (for one or more DCM connectivity parameters) appear to be necessary to explain between window differences. Note that Park et al. (2017) did not include an exponential decay function in their analysis. In the present dataset, subjects were asked to keep their eyes open for 1 min, which was then followed by short block in which they were instructed to keep their eyes closed – and other movement task blocks). Therefore, subjects in our study might still be in a sort of adaptation phase during acquisition of the data. Park et al. (2017), on the other hand, used data from the Human connectome project, in which participants are asked to relax inside the MRI scanner for approximately 15 min. This difference in context might explain why our findings differ from Park et al. (2017).

Considering the group level parameters in Fig. 5, Fig. 6 – and the group level estimates of the connectivity trajectory of both networks (Fig. 7) – the similarities are apparent. In both networks, we see that most (log-scaling) connections are negative for both the forward and backward connections while positive effect can be seen for the lateral connections. A reversed exponential decay for the forward connection is apparent in both networks. Looking at the group level trajectory, both networks give qualitatively similar temporal effects with respect to the forward connections.

As noted above, negative log-scaling parameters mean that the fixed values corresponding to excitatory connections in the neural model are downscaled by the scaling parameter. In the neural mass model we used here, forward connections have a strong driving effect, while backward connections have more inhibitory and modulatory influence on the nodes they target. This difference in forward and backwards connections implies a functional cortical hierarchy. In light of our results, connectivity from low to higher order brain regions becomes more pronounced. This suggests that more information is conveyed through the network with time in ascending or bottom-up fashion during this first minute of rest eyes-open period.

Given the similarities of the group level trajectories between the networks, these results appear to be at odds with the results of the Bayesian model comparison of second level models. More specifically, the absence of significant DCT components at the group level seems to be at odds with the BMS suggesting a contribution of some DCT components at the within-subject level. This apparent discrepancy can be resolved by considering that the PEB treats the DCM parameters (or second level parameters for the PEB of PEB) as random effects, with mean equal to (X⊗I)β Therefore, although at the individual subject level, some DCT components show significant effects; these are not conserved over subjects. In other words, they have a group mean of zero. This also means that the trajectories are unique for each individual. Only the decay component embedded in the parameter trajectories is conserved over subjects. For the saliency mode network however, the DCT effects at the individual subject level are not systematically different from zero (except for the lateral connections). A possible explanation might be that subject specific factors – such as age, gender, and fatigue – had a larger effect on the default mode trajectories compared to the saliency network. For the saliency, the effect might be mainly driven by the context, which is common to all subjects. In sum, we showed that connectivity trajectories can contain components that are subject-specific but systematic (e.g. DCT components) and components which are conserved over subjects (e.g. monotonic increase in both networks).

In the present work, we chose a window size of 1 s. However, window size can have a substantial effect on the connectivity estimates – and the use of sliding windows in general has been criticised (e.g. Hlinka and Hadrava, 2015; Laumann et al., 2017; Leonardi & Van De Ville, 2015). One of the main points raised – against the use of short windows – is that estimates of connectivity are less efficient and would therefore inflate the apparent variability in connectivity over time. In classical analyses, one can mitigate against this using an appropriate null model (Hlinka and Hadrava, 2015; Laumann et al., 2017; Lindquist et al., 2014). In our study, the null model can be regarded as the second level (reduced) model containing only the constant term (i.e. model 16). Having said this, in Bayesian analyses, the loss of efficiency with short windows leads to an *increase in the posterior variance* – not an increase in the *variance of the posterior estimate*. This means that our DCM analysis is immune from the problem of short windows: note that the second level models include random fluctuations about smoothly varying parameters, whose estimated amplitude depends upon the posterior variance from the first level. Using Bayesian model comparison, we have shown that second level models with systematic fluctuations in connectivity (modelled by temporal basis functions) outperform the null model – in terms of the accuracy-complexity trade-off (i.e., free energy). In the context of functional connectivity, it has been shown that spurious time-varying connectivity can arise due to the sliding window approach (Leonardi & Van De Ville, 2015). Leonardi & Van de Ville suggest that the minimum window size should at least be as large as the period of the lowest frequency component in the resting state data. In our analyses, all data features below 1Hz were high-pass filtered. It should be noted that choosing longer windows increases the risk of violating any stationarity assumption. For example, Park et al. (2017) demonstrated increased consistency of connectivity estimates, when using time-varying models compared to non-time-varying models, illustrating the importance of modelling time-varying connectivity. In the context of fluctuations in effective connectivity, one can choose the window length to ensure efficient parameter estimation. This generally requires simulations – in which the ground truth is known – to evaluate the minimum window length required in a particular situation (e.g., size of network, number of channels *etc*.). In the setting of DCM for rs-fMRI, this approach has been considered by (Razi et al., 2015). For EEG however, the choice of window length has yet to be addressed in a principled way. In principle, it should be possible to optimise window length using Bayesian model comparison. This is because a partition of the timeseries into windows does not change the data; therefore, the window length can be regarded as an attribute of the hierarchical model. An alternative approach would be to examine the dependency of the information gain (as scored by the KL divergence between priors and posteriors) on window length. We hope to pursue this in future work.

Given the non-invasive nature, relative low cost and portability of EEG, tracking time-variability of connectivity can be readily applied in patient studies. Deriving connectivity fluctuations from EEG might be a promising avenue for future research. Differences in parameter trajectories could be used as a neural signature of pathophysiology or psychopathologies (see e.g. Bolton et al., 2018, for an example using fMRI). Also, treatment effects could be related to connectivity fluctuations. Another possible application of the approach is to model factors such as fatigue, motivation, and learning over the course of an experimental procedure. This would require dividing the experiment, *post-hoc*, in a number of blocks in which trials are averaged (i.e. creating ERP's). Although this would reduce the signal to noise ratio of the ERP's within blocks, modelling such factors might outweigh the cost. For example, it could be that the mismatch negativity, a brain response to a violation of a rule, is reduced towards the end of an experiment. Using hierarchical modelling one could then disambiguate between e.g. low level reduction in sensitization and reduced top-down influences (see Garrido et al., 2009 for a similar approach). To conclude, our study showed that effective connectivity showed both common (saliency network and default mode network) and subject-specific (saliency network) trajectories over the course of 1 min rest EEG.
