# Supplementary material for: Dynamic causal modelling of fluctuating connectivity in resting-state EEG
Source: Neuroimage. 2019 Apr 1;189:476–84. doi: 10.1016/j.neuroimage.2019.01.055 (PMC6435216; doi:10.1016/j.neuroimage.2019.01.055)
Supplement: Multimedia component 1 [file mmc1.docx]

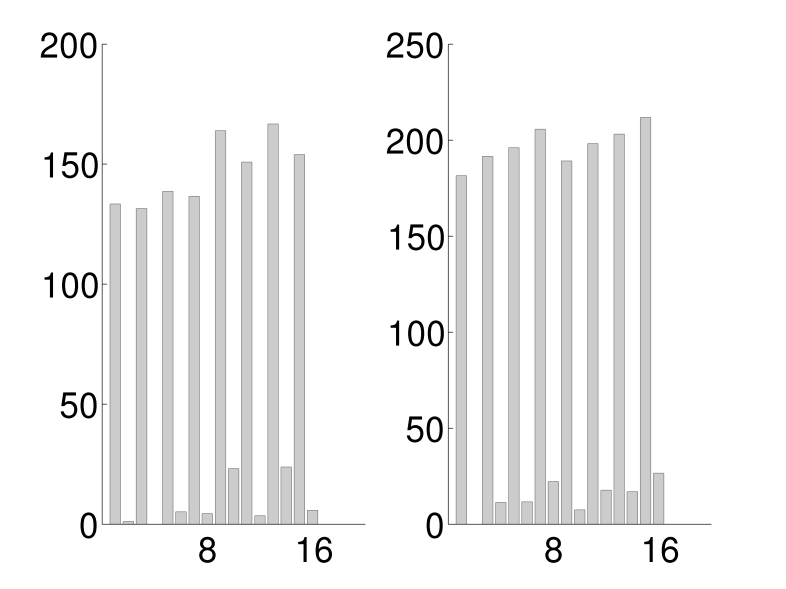


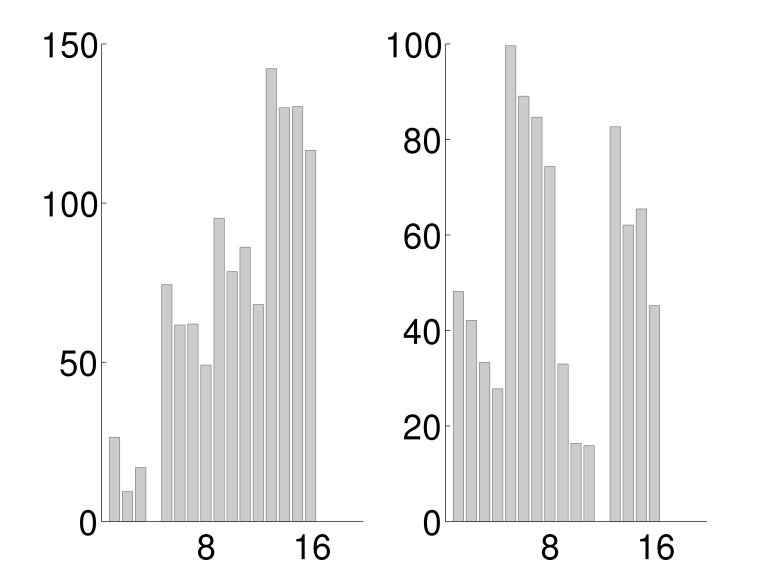


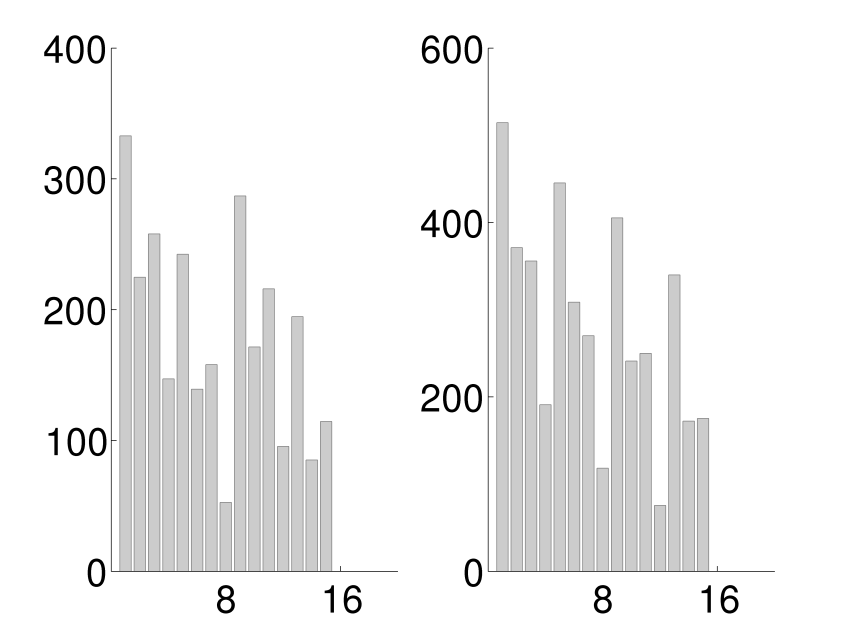


Figure S1: The BMC results for the split-groups are shown fort the default mode network. Top, middle and bottom panels shows the forward backward and lateral connections respectively. Left and right plot are the results for the first and second subgroup.


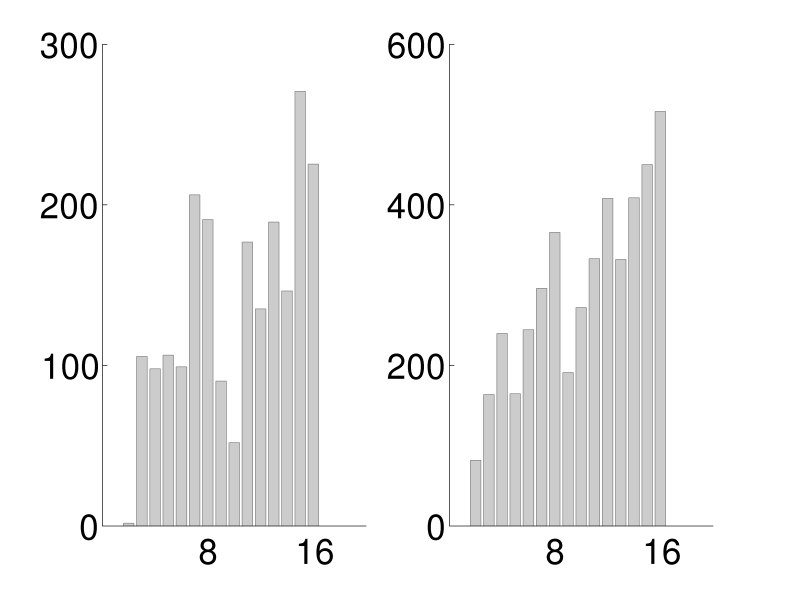


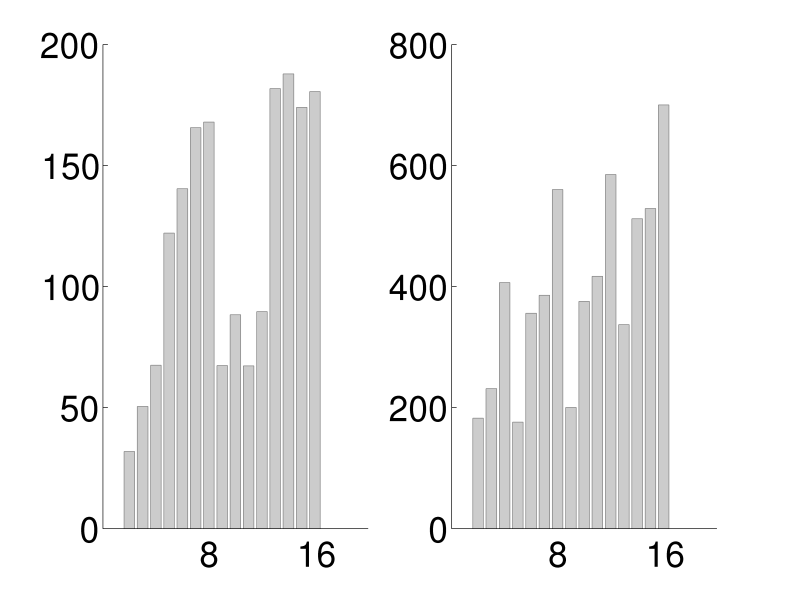


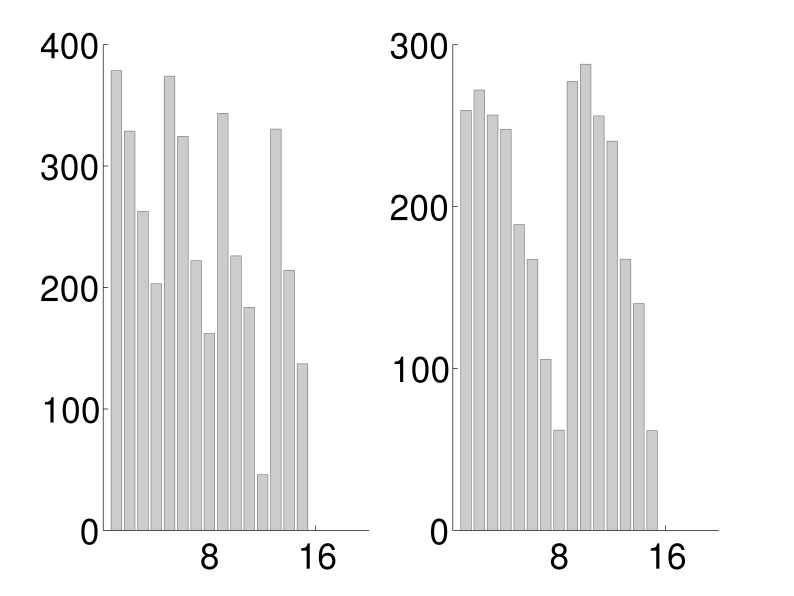


Figure S2. Same format as fig. S1 but for the saliency network.


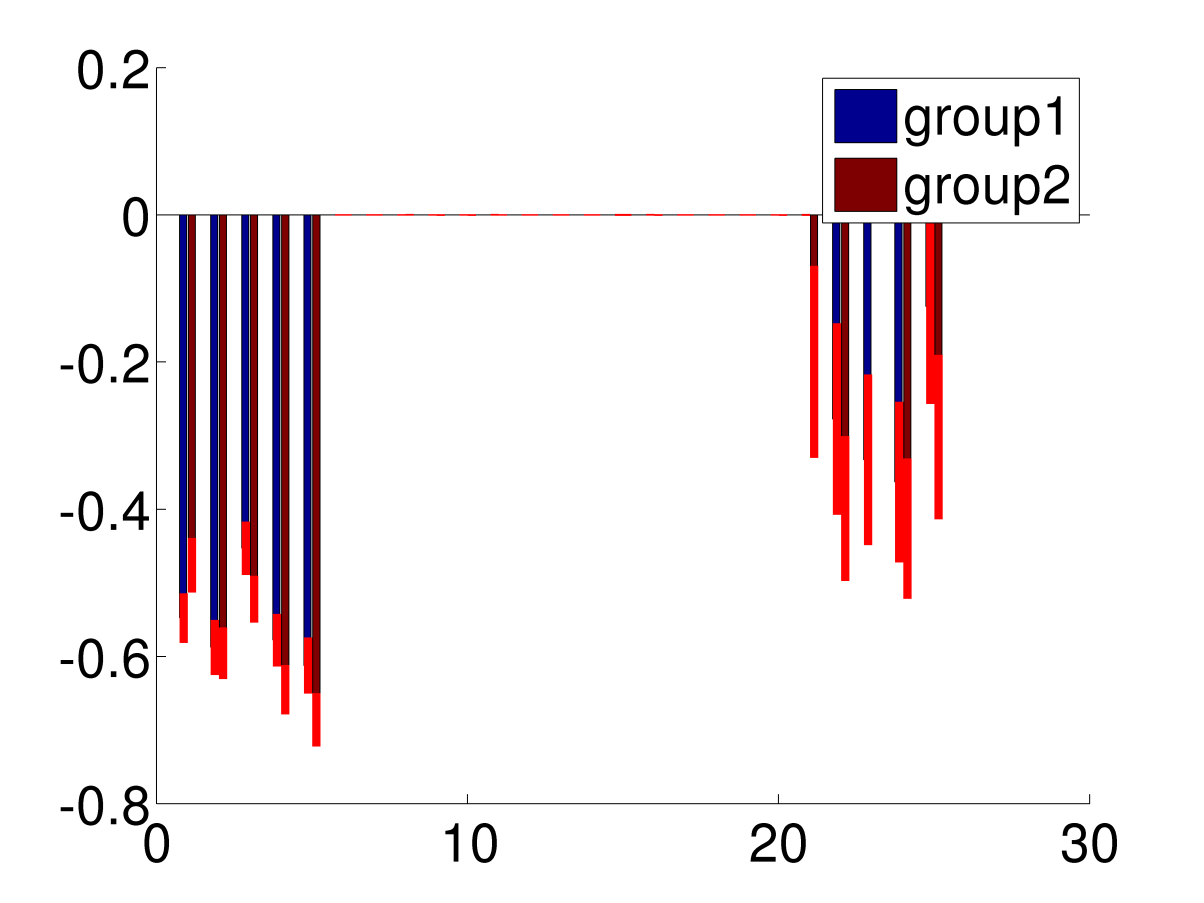

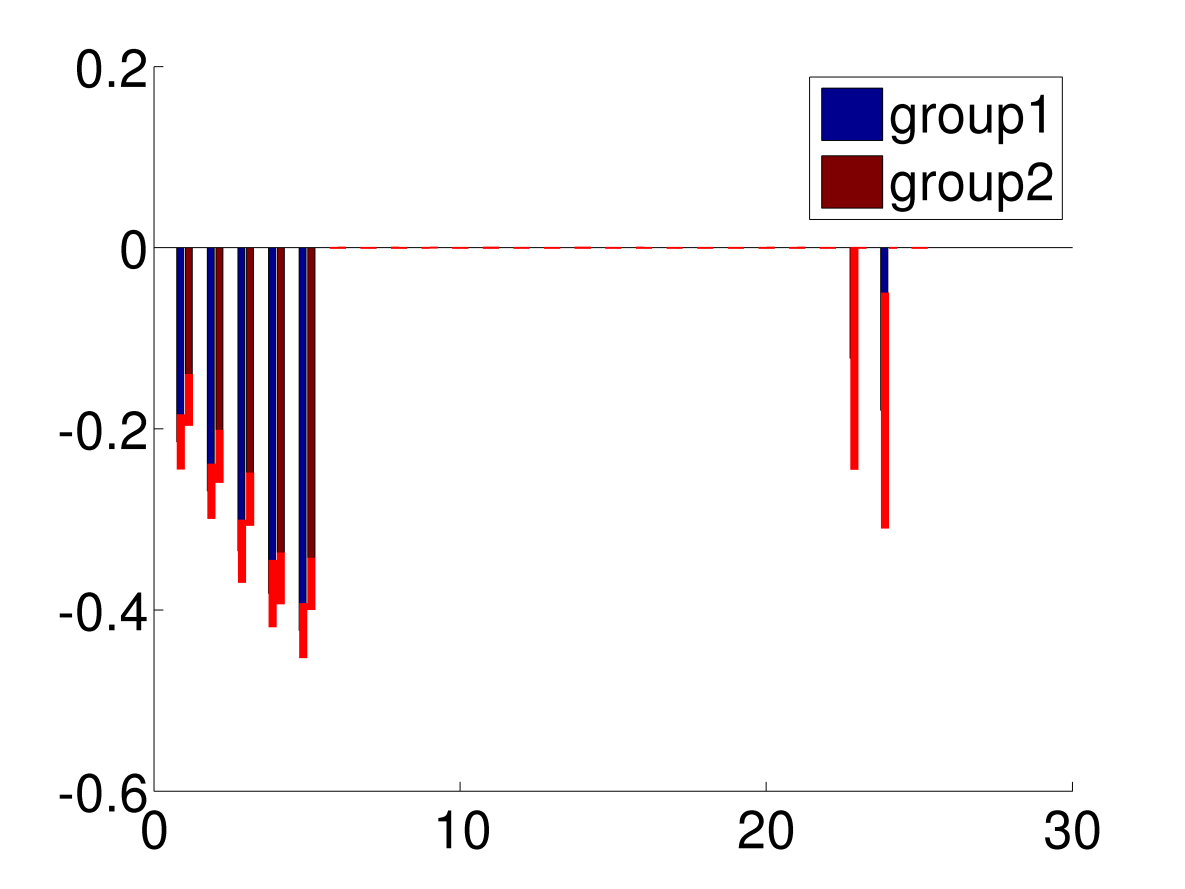

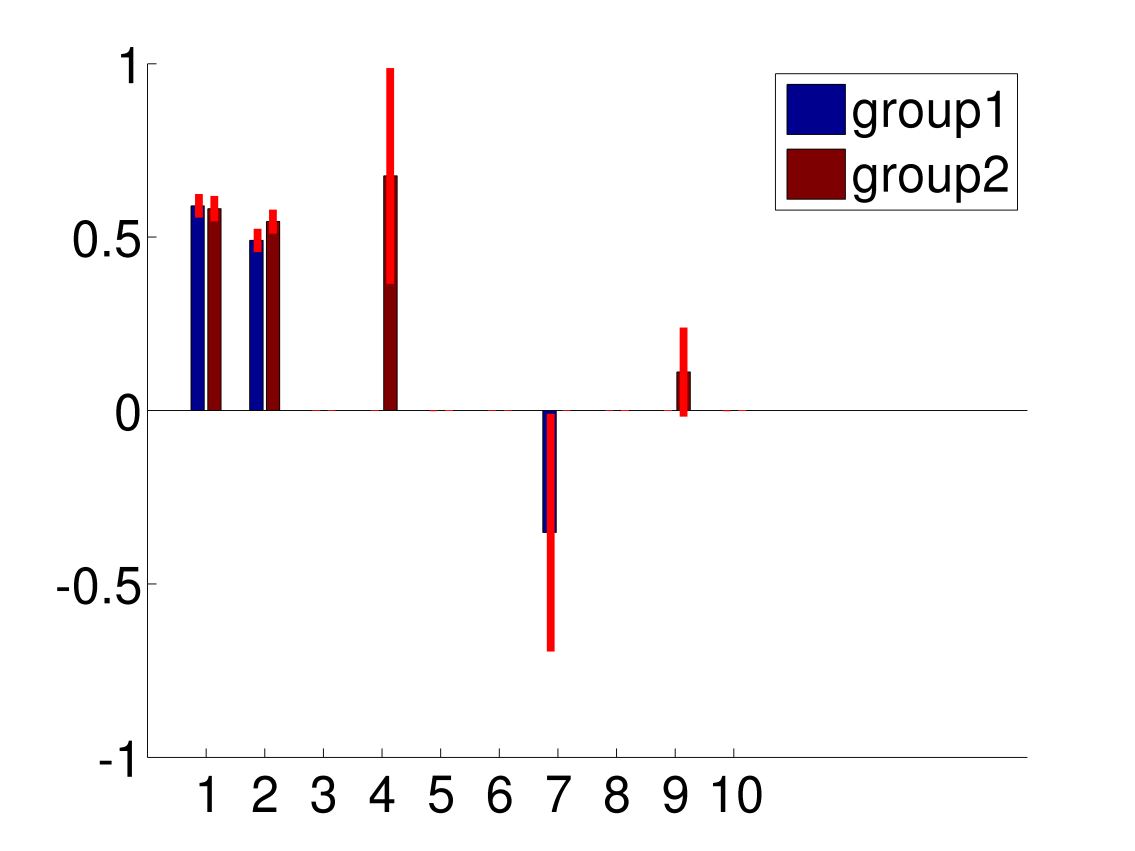


Figure S3. The results of the group PEB conducted on the two subgroups, following BMA for the default mode network. Top, middle and bottom panels shows are the BMA parameters of the forward, backward and lateral connections respectively.


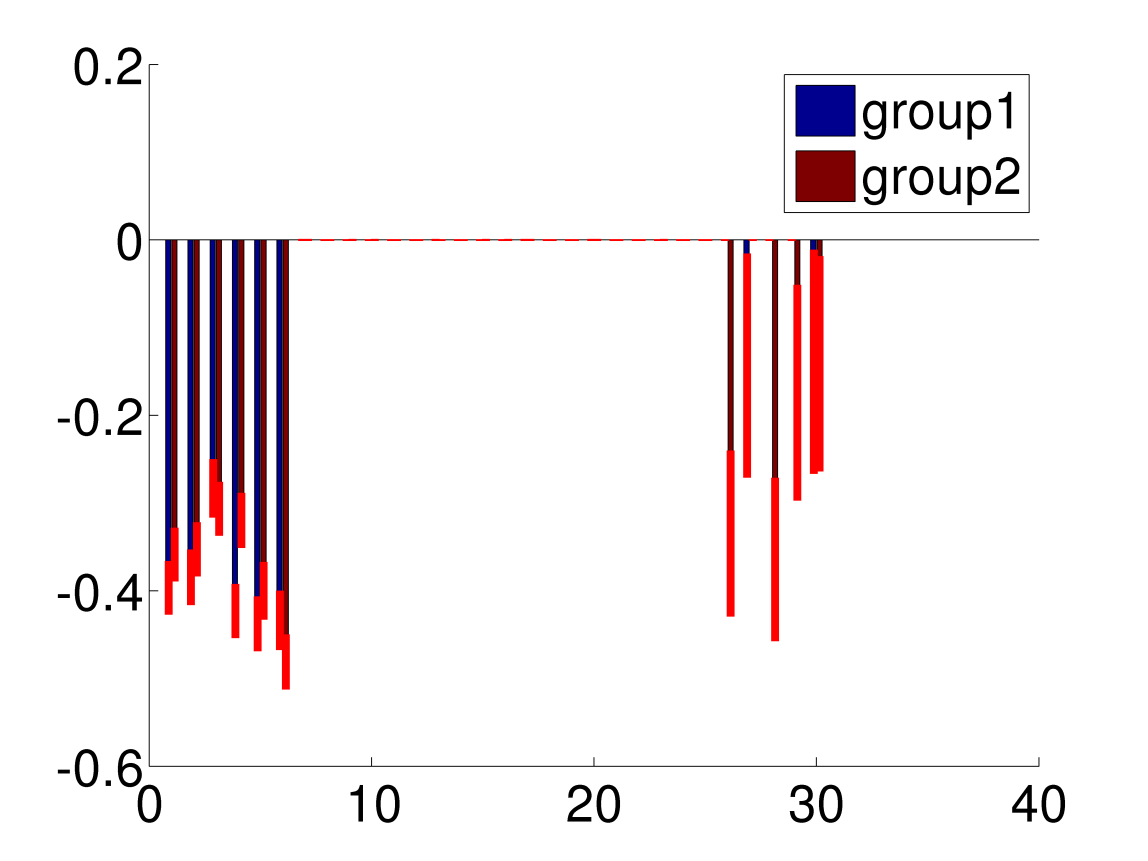

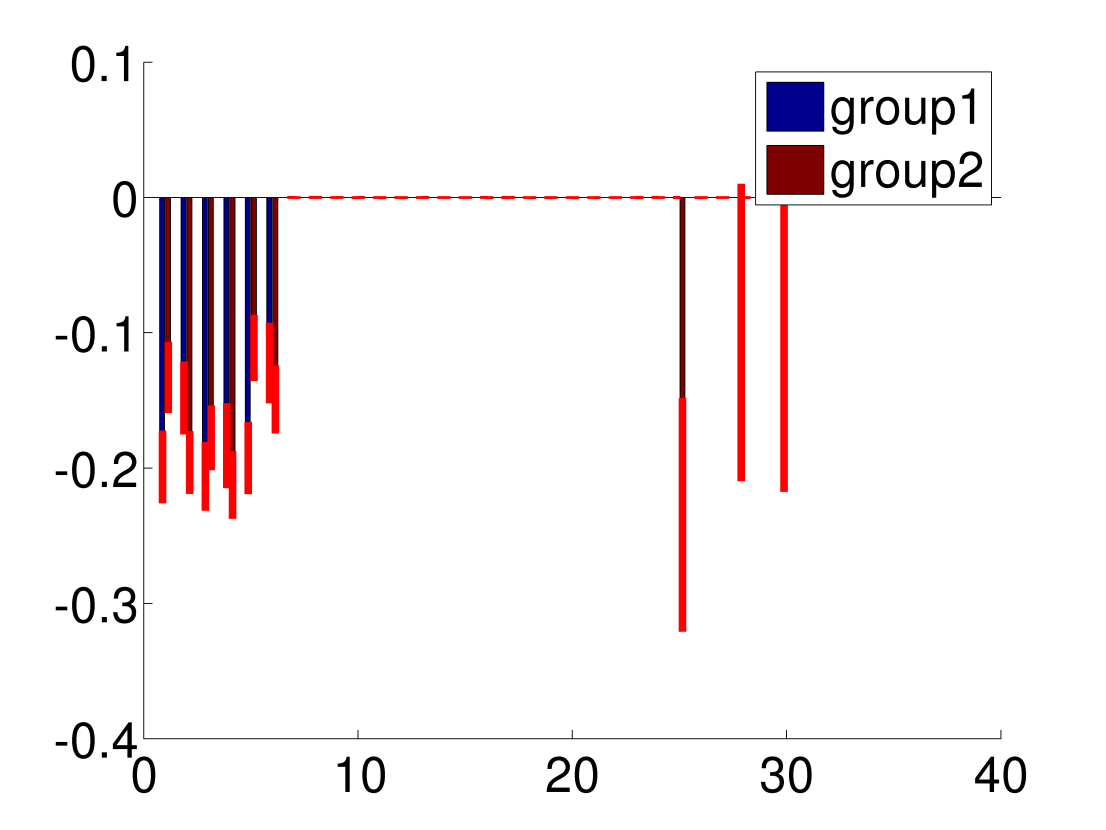

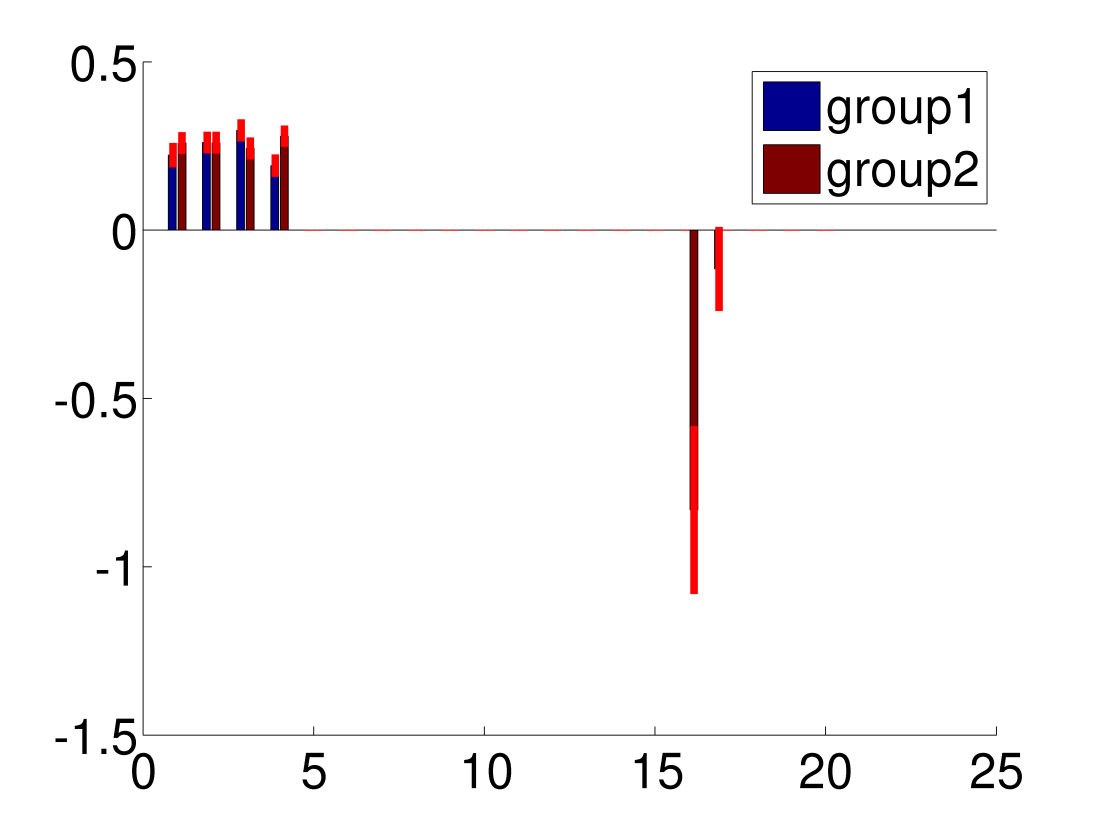


Figure S4. Same format as S3 but for the saliency network.


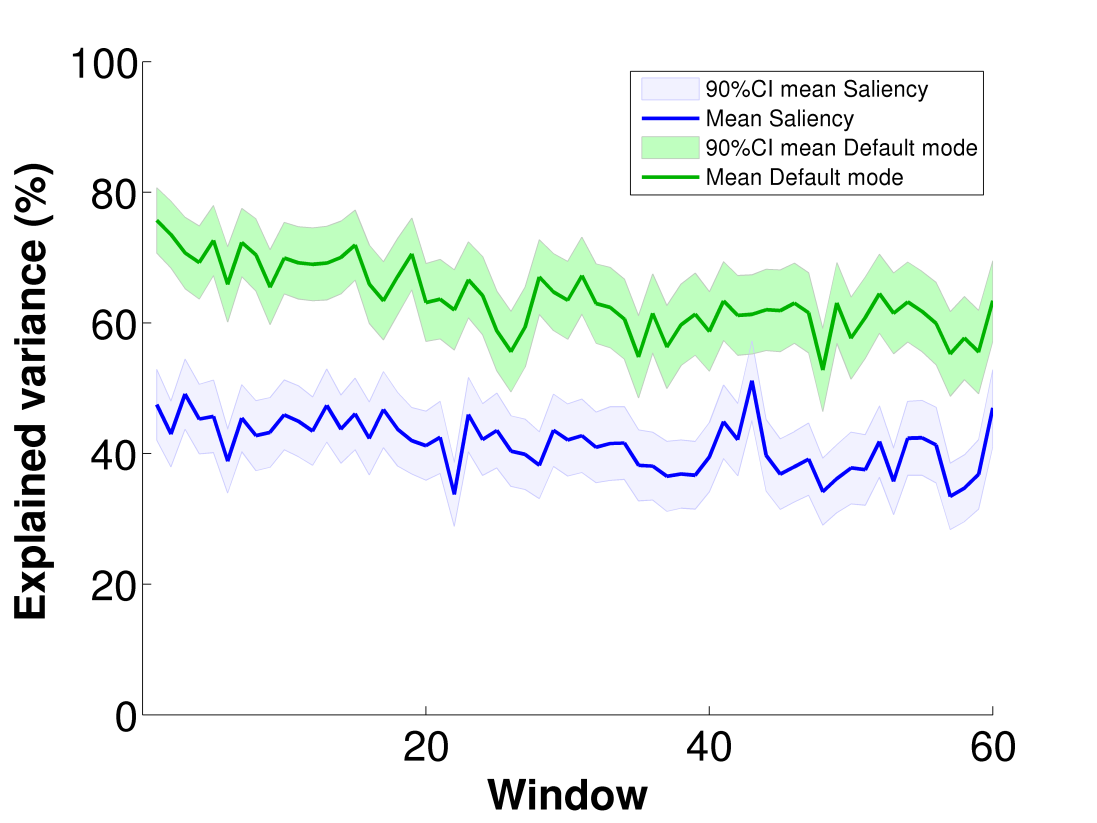


Figure S5. Average explained variance as a function of time window together with a 90% confidence interval about the mean for the default mode network (green) and saliency network (blue)
